# Worry from contracting COVID-19 infection and its stigma among Egyptian health care providers

**DOI:** 10.1186/s42506-021-00099-6

**Published:** 2022-01-10

**Authors:** Doaa Mohamed Osman, Fatma R. Khalaf, Gellan K. Ahmed, Ahmed Y. Abdelbadee, Ahmed M. Abbas, Heba M. Mohammed

**Affiliations:** 1 Department of Public Health and Community Medicine, Faculty of Medicine, Assiut University, Assiut, Egypt; 2 Family and Community Health Nursing, Faculty of Nursing, Assiut University, Assiut, Egypt; 3 Department of Neurology and Psychiatry, Faculty of Medicine, Assiut University, Assiut, Egypt; 4 Department of Child and Adolescent Psychiatry, Institute of Psychiatry, Psychology and Neuroscience, King’s College London, London, UK; 5 Department of Obstetrics and Gynecology, Faculty of Medicine, Assiut University, Assiut, Egypt

**Keywords:** Health care providers, Egypt, Worry, Stigma, COVID-19

## Abstract

**Backgrounds:**

Healthcare providers (HCPs) in COVID-19 epidemic face stressful workload of disease management, shortage of protective equipment and high risk of infection and mortality. These stressors affect greatly their mental health. The aim is to identify working conditions among Egyptian HCPs during COVID-19 epidemic as well as stigma and worry perceptions from contracting COVID-19 infection and their predictors.

**Methods:**

A cross-sectional study was conducted among 565 HCPs. Data was collected through Google online self-administered questionnaire comprised seven parts: demographics characteristics, knowledge and attitude of COVID-19, working condition, worry of contracting COVID-19 at work, discrimination intention at work for COVID-19 patients, stigma assessment using impact stigma, and internalized shame scales.

**Results:**

The vast majority of HCPs (94.7%) were worried from contracting COVID-19 at work. Risk factors for perceiving severe worry from contracting COVID-19 were expecting infection as a severe illness, believing that infection will not be successfully controlled, improbability to continue working during the pandemic even if in a well/fit health, high discrimination intention and impact stigma scales. Significantly high impact stigma scores were detected among those aged < 30 years, females, workers primarily in sites susceptible for contracting COVID-19 infection, those had severe worry from contracting infection at work, and high internalized shame scale. The risk factors for perceiving higher internalized shame scores were not having a previous experience in working during a pandemic, high discrimination intention towards COVID-19 patients and high impact stigma scale.

**Conclusions:**

Considerable levels of worry and stigma were detected among Egyptian HCPs during COVID-19 outbreak. The psychological aspect of health care providers should not be overlooked during epidemic; appropriate institutional mental health support should be provided especially for young HCPs, those without previous work experience in epidemic and those who work in high-risk units. Raising the community awareness about contribution of HCPs in fighting the epidemic might decrease stigmatization action toward HCPs.

## Introduction

Since the announcement of the Chinese authorities in Wuhan City on the novel coronavirus (COVID-19) in December 2019, the epidemic has expanded from Wuhan throughout China and disseminated to every country across the world [1]. As of 30 November, 2021 there are 261.5 million confirmed cases of COVID-19, including 5.2 million deaths worldwide [2].

COVID-19 infection occurs mainly by contact or droplet transmission. In addition, airborne infection may develop in case of respiratory aerosols production with patient respiratory activity or medical procedures [3].

Similar to the preceding communicable disease epidemics, healthcare workers in COVID-19 epidemic are at the front line in terms of risk of infection and mortality [4]. Health care systems worldwide have become overburdened with possibly infectious patients seeking testing and health care. Personal protective equipment (PPEs) are important during the current COVID-19 pandemic [3]. A critical lack of protective equipment is predicted to occur or has already occurred in high demand areas [5].

The rapid spread of COVID-19 infection across the world caused a degree of fear and worry in the general population and specifically among certain groups as elderly, people with underlying health conditions and health care providers (HCPs) **[**6**]**. During epidemics, the mental health of HCPs is seriously challenged. In the early phase, psychological distress as fear and anxiety appear then gradually decline. Later on, depression, and posttraumatic stress symptoms appear and persist for a long time, leading to considerable impact. HCPs isolation, working in high-risk sites, and dealing with infected patients are the main reasons of trauma. Moreover, some HCPs may experience avoidance by their family or community **[**7, 8**]**.

In Egypt, the first case of COVID-19 was confirmed on 14 February 2020 [9], then there was growing rise in the number of confirmed cases and deaths. Concurrently, there was a parallel rise in the number of infected HCPs including physicians and nurses. This study aimed to identify the working conditions among Egyptian HCPs during COVID-19 epidemic as well as the perception of stigma and worry from contracting this infection and their predictors.

## Methods

### Study design and population:

An online survey was conducted on 565 Egyptian HCPs. HCPs were physicians, nurses, and health officers (both medical and nursing specialty).

### Sample size and technique:

The sample size was estimated using the EPI info statistical package, Version 7. The used parameters were a proportion of 0.5, a confidence level of 95% and a margin of error of 5%. Sample size was 384 HCPs and 10% as non-response rate was added. Non-probability convenience sampling technique was applied. Data was collected using Google online self-administered questionnaire disseminated in Arabic language. The questionnaire link was distributed using social networks through either personal communication or physician/nurses gathering groups. Data was collected from 24th April till 7th May 2020.

### The used tool

The self-administered questionnaire consisted of seven parts:

1. Demographics characteristics: age, gender, marital status, education, occupation, years of experience, and residence either urban/rural and governorate.
2. Knowledge about COVID-19 included 12 questions, each question had three answer options as true, false and “I do not know.” One point was assigned for each correct answer while zero point was assigned for the incorrect or unknown answer [10].
3. Attitude toward control of COVID-19 was inquired by two questions. Whether he/she agree that COVID-19 will finally be successfully controlled, and if he/she has the confidence that Egypt can control the epidemic? [10, 11]
4. Working conditions during epidemic were assessed using red caps questionnaire for health care providers about COVID-19 [12].
5. One statement assessed worry of contracting COVID-19 at work since announcement of the first COVID-19 case in Egypt. The response categories were not at all worried, somewhat worried, and very worried [13].
6. Discrimination intention at work for COVID-19 patients was measured. Discrimination intention was assessed using four items each item scored from 1 (strongly disagree) to 5 (strongly agree) point. The total score ranged from 4 points to 20 points. Higher scores meant less discrimination intent toward COVID-19 patients [14]. The reliability of the discrimination intention scale assessed by using Cronbach’s alpha was 0.959.
7. Two scales were used to assess stigma perception by HCPs due to their dealing with COVID-19 patients. The questions were derived from scales used to assess stigma from dealing with patients living with HIV as a serious disease [15], each item was scored from 1 (strongly disagree) to 5 (strongly agree) point and higher scores indicated more perception of stigma. The first scale measured the perceived impact of working with COVID-19 or its related field. The second was the Internalized Shame Scale measured how the providers felt about their work with COVID-19 patients. The reliability of the impact and internalized shame scales assessed using Cronbach’s alpha were 0.802, 0.685 respectively.

### Statistical analysis

Data was analyzed using the Statistical Package for Social Science (SPSS), version 16.0 for Windows. Mean and standard deviation were used to express quantitative data while qualitative data was presented using frequencies and percentages. Unadjusted and adjusted logistic regression models were applied to identify predictors of being very worried from contracting COVID-19 at work as a dependent variable (very worried = 1 and somewhat worried/not worried at all = 0). Unadjusted and adjusted linear regression models were used to identify predictors of stigma both impact and internalized shame scales. The explanatory variables entered in the adjusted regression models were age and gender and the significant variables resulted from the unadjusted models. Significance of statistical tests was at *P* value < 0.05.

## Results

Table 1 shows the sociodemographic characteristics of the studied HCPs (*n* = 565) were as follows: participants aged 25 to 40 years old were 69.4%. Females represented 70.6% of the participants, urban residents formed more than three quarters (77.3%) and the majority (95.4%) were residing in Upper Egypt. More than half of the participants were physicians (53.6%), while nurses formed 22.7%. Concerning work experience duration, the highest percentage of providers had more than 6 years’ experience (45.8%). Married subjects were 51.5%. Among ever-married subjects (*n* = 302), 86.1% had children.

**Table 1:** Socio-demographic characteristics of the studied health care provider, Egypt (2020)

Variable	***n*** = 565	%
**Age**		
Less than 25	108	19.1
25–30	204	36.1
30–40	188	33.3
> 40	65	11.5
**Gender**		
Male	166	29.4
Female	399	70.6
**Residence**		
Urban	437	77.3
Rural	128	22.7
**Governorate**		
North upper Egypt	181	32.0
Middle upper Egypt	209	37.0
South upper Egypt	149	26.4
Others	26	4.6
**Occupation**		
Health officer	109	19.3
Nursing officer	25	4.4
Physician	303	53.6
Nurse	128	22.7
**Years of experiences**		
< 2 years	173	30.6
2–<4 years	72	12.7
4–6 years	61	10.8
> 6 years	259	45.8
**Marital status**		
Single	263	46.5
Married	291	51.5
Widow/divorced	11	2.0
**Having children(n=302)**		
Yes	260	86.1
No	42	13.9

Table 2 shows the working conditions and personal perceptions of COVID-19. Forty percent of the studied HCPs were primarily working in sites with higher probability for contracting COVID-19 infection. Nearly one-fifth (19%) had an experience in working during previous pandemics. Those reported exposure to even one diagnosed or suspicious COVID-19 patient were 31.7%. About 35% received training on COVID-19, 71.5% received updated guidelines on occupational safety and 22.3% of the participants reported that their institutions provided psychological/mental health support. The majority of studied subjects applied changes in their personal protection after announcement of COVID-19 cases in Egypt. Nearly 70% intended to continue work during COVID-19 pandemic if they were fit/in well health. The majority perceived COVID-19 as severe disease and making their loved ones sick and becoming sick were the main personal worries of the participants. The total COVID-19 knowledge score ranged from 6 to 12 with a mean of 10.92 ± 0.92. Those who agreed that COVID-19 pandemic will be successfully controlled were 73.8% and 47.6% were confident that Egypt can control the epidemic. The majority of Egyptian HCPs were worried from contracting COVID-19 at work; either very worried (35.6%) or somewhat worried (59.1%).

**Table 2:** Working conditions and personal perceptions of COVID-19 among the study health care providers, Egypt (2020)

Variables	***n*** = 565	%
**Whether primarily work in ED, ICU, or infectious diseases dep.**		
Yes	225	39.8
No	340	60.2
**Having direct experiences in working during a previous pandemic**		
Yes	108	19.1
No	457	80.9
**Exposure to at least one diagnosed/suspicious COVID-19-infected person**		
Yes	179	31.7
No	386	68.3
**Receiving any training about COVID-19**		
Yes	196	34.7
No	369	65.3
**Receiving appropriate guidelines on updated procedures for personal safety to be followed at work**		
Yes	404	71.5
No	161	28.5
**Availability of institutional psychological/mental health support**		
Yes	126	22.3
No	439	77.7
**Being redirected to COVID-19-related activities**		
Yes	161	28.5
No	404	71.5
**Applied changes in personal protection after COVID-19 hit Egypt** #		
Started routinely wearing masks	430	76.1
Started using hand sanitizer/more frequent hand washing	531	94.0
Started wearing scrubs/disposable clothes	154	27.3
Started social distancing	466	82.5
**Deciding not to wear a mask or other PPE** #		
No never	476	84.2
Yes, because none of my colleagues was wearing it	4	0.7
Yes, because my employer did not recommend it	11	1.9
Yes, because it is uncomfortable/inconvenient	38	6.7
Yes, because I do not believe I need to wear it	29	5.1
Yes, because it is not available	18	3.2
**Probability of work continuation during COVID-19 pandemic if they are fit/well**		
Extremely unlikely	38	6.7
Unlikely	31	5.5
Neutral	97	17.2
Likely	195	34.5
Extremely likely	204	36.1
**Personal perception of severity of COVID-19**		
Not severe	25	4.4
Neutral	76	13.5
Simple	5	0.9
Severe	459	81.2
**Personal worries about working during COVID-19 pandemic** #		
Becoming sick	311	55.0
Making my loved ones sick	538	95.2
Not having the appropriate skills to care for COVID-19 patients	200	35.4
Not having adequate supplies to care for COVID-19 patients	260	46.0
Others^$^	18	3.2
**Worry from contracting COVID 19 at work**		
Very worry	201	35.6
Somewhat worry	334	59.1
Not worried at all	30	5.3

*ED* emergency department, *ICU* intensive care unit, *COVID-19* coronavirus disease-2019, *PPE* personal protective equipment

^#^Multiple answers were allowed.

^$^Others (there is no vaccine, have no protective equipment, in case of death no compensation will be provided for the family)

Nearly half of participants had no discrimination intents towards management of COVID-19 cases either working with COVID-19 patients, providing care, performing physical examination and interacting with COVID-19 patients similar to non-COVID-19 cases. Due to their work with COVID-19 patients or its related field, nearly 40% of participants suffered discrimination or stigma outside work or noticed that people were moving away from them at  some social functions and 54.4% reported unhappiness of their families. On the other hand, lower percentages reported reduction of contact by friends (28.2%) and suffering discrimination or stigma from their colleagues (16.6%) due to the same cause. About one-fifth of the studied health care providers felt embarrassed to tell people about their work nature and wished if they could change their occupation or would never deal with COVID-19 patients. The mean of the discrimination intent scale was 12.04 ± 4.42, while impact scale was 13.90 ± 4.18 and internalized shame scale was 4.62 ± 1.97 (Table 3).

**Table 3:** Discrimination intent and stigma (impact and internalized shame scales) among the studied health care providers, Egypt (2020)

Scale	Strongly disagree	Disagree	Neither agree nor disagree	Agree	Strongly agree
**Discrimination intent scale**					
Willing to work with COVID-19 patients	88 (15.6)	120 (21.2)	85 (15.0)	260(46.0)	12 (2.1)
Willing to provide same care as other patients	75 (13.3)	114 (20.2)	83 (14.7)	279 (49.4)	14 (2.5)
Willing to do physical exam of COVID-19 patient as other patients	85 (15.0)	126 (22.3)	73 (12.9)	271 (48.0)	10 (1.8)
Willing to interact same as other patients	91 (16.1)	113 (20.0)	78 (13.8)	269 (47.6)	14 (2.5)
Mean ± SD (range)	12.04 ± 4.42 (4.00-20.00)				
**The perceived stigma using impact scale: Since announcement of COVID-19 cases in Egypt**					
Suffering discrimination or stigma outside work due to working in COVID-19-related field.	69 (12.2)	186 (32.9)	80 (14.2)	207 (36.6)	23 (4.1)
Friends decreased their contact with you since you started working with COVID-19 patients or its related field.	105 (18.6)	214 (37.9)	87 (15.4)	144 (25.5)	15 (2.7)
People move away from you at social functions when hearing that you work with COVID-19 patients or its related field.	54 (9.6)	164 (29.0)	94 (16.6)	228 (40.4)	25 (4.4)
Unhappiness of family due to working with COVID-19 patients or its related field.	54 (9.6)	124 (21.9)	80 (14.2)	262 (46.4)	45 (8.0)
Suffering discrimination or stigma from other staff at the hospital due to the work with COVID-19 patients or its related field.	134 (23.7)	256 (45.3)	81 (14.3)	86 (15.2)	8 (1.4)
Mean ± SD (range)	13.90 ± 4.18 (5.00–20.00)				
**Internalized shame scale**					
If you worked with COVID-19 patients, you would feel embarrassed to tell other people about it.	161 (28.5)	226 (40.0)	61 (10.8)	104 (18.4)	13 (2.3)
If you worked with COVID-19 patients, you would wish that you could change your job so that you would never have to deal with COVID-19 patients.	136 (24.1)	226 (40.0)	81 (14.3)	103 (18.2)	19 (3.4)
Mean ± SD (range)	4.62 ± 1.97 (2.00–10.00)				

*COVID-19* coronavirus disease-2019, *SD* standard deviation

Table 4 displayed the risk factors of worrying from contracting COVID-19 infection at work among studied health care providers. In the adjusted model, the significant predictors were perceiving COVID-19 as severe disease (OR = 1.99; *P* = 0.011), believing that COVID-19 infection will not be successfully controlled (OR = 1.70; *P* = 0.033), improbability to continue working during the COVID-19 pandemic even in a well/fit health (OR = 1.88; *P* = 0.005). In addition, high discrimination intention towards COVID-19 patients (as low scores indicate high discrimination intention) (OR = 0.88; *P* < 0.001) and perceiving stigma resulted from actions of surrounded subjects using impact scale (OR = 1.08; *P* = 0.003) were risk factors.

**Table 4:** Predictors of severe worry of contracting COVID-19 infection at work among the studied health care providers, Egypt (2020)

Variable	Unadjusted logistic regression			Adjusted logistic regression		
	Odds ratio	95% CI	***P*** value	Odds ratio	95% CI	***P*** value
**Age (< 30 years)**	1.55	1.09–2.21	**0.01**	1.30	0.87–1.96	0.200
**Gender (female)**	1.16	0.79–1.70	0.43	0.88	0.57–1.37	0.572
**Marital status (married)**	1.22	0.86–1.726	0.25			
**Occupation (doctors)**	1.19	0.80–1.76	0.38			
**Years of experience (< 4 years)**	1.36	0.96–1.92	0.08			
**Primarily work in ED, ICU, or infectious diseases dep. (yes)**	1.29	0.90–1.83	0.15			
**Experience in working during a previous pandemic (no)**	1.47	0.93–2.32	0.09			
**Exposure to at least one person who has been diagnosed or had symptoms suggestive of COVID-19 infection (yes)**	0.81	0.56–1.18	0.28			
**Receiving any training about COVID-19 (no)**	1.21	0.84–1.75	0.29			
**Receive appropriate guidelines on updated procedures related to personal safety to follow at work (no)**	1.85	1.27–2.69	**0.001**	1.27	0.81–1.98	0.293
**Employer provision of adequate PPE (no)**	1.93	1.36–2.74	**< 0.001**	1.23	0.81–1.87	0.325
**Availability of institutional psychological/mental health support (no)**	1.50	0.97–2.31	0.06			
**Being redirected to activities related to COVID-19 (yes)**	0.95	0.65–01.39	0.80			
**Personal perception of COVID-19 (severe disease) (severe)**	1.78	1.11–2.87	**0.017**	1.99	1.17–3.39	**0.011**
**Thinking that COVID-19 will finally be successfully controlled (no)**	2.56	1.74–3.76	**< 0.001**	1.70	1.04–2.76	**0.033**
**Confidence that Egypt can control the epidemic (no)**	2.25	1.58–3.22	**< 0.001**	1.43	0.91–2.25	0.119
**Underestimating dangerous effect of COVID-19 on general health in its early days (no)**	1.48	1.04–2.10	**0.02**	1.30	0.88–1.93	0.188
**Probability of work continuation during COVID-19 pandemic if she/he is fit/well? (unlikely)**	2.96	2.03–4.31	**< 0.001**	1.88	1.21–2.91	**0.005**
**Knowledge score**	0.97	0.81–1.77	0.80			
**Discrimination intent score**	0.85	0.81–0.88	**< 0.001**	0.88	0.84–0.93	**< 0.001**
**Impact stigma score**	1.10	1.05–1.15	**< 0.001**	1.08	1.03–1.14	**0.003**
**Internalized shame scale**	1.29	1.17–1.413	**< 0.001**	1.03	0.92–1.16	0.560

*ED* emergency department, *ICU* intensive care unit, *COVID-19* coronavirus disease-2019, *PPE* personal protective equipment

Bold values: statistically significant at *P* value < 0.05

Table 5 displayed the adjusted and unadjusted linear regression models for identifying risk factors for perceiving stigma resulted from actions of surrounded subjects (impact scale) among HCPs. In the adjusted model, the significant predictors were age less than 30 years, being female, primarily working in sites lending them susceptible for contracting COVID-19 infection, severe worry from contracting COVID-19 infection at work, and high perception of internalized shame from dealing with COVID-19 patients or working in its related field (*P* < 0.05).

**Table 5:** Predictors of stigma (impact scale) among the studied health care providers, Egypt (2020)

Variable	Unadjusted linear regression			Adjusted linear regression (***R***^**2**^ = 0.19)		
	ß	95% CI	***P*** value	ß	95% CI	***P*** value
**Age (< 30 years)**	1.31	0.62–1.99	**< 0.001**	1.05	0.41–1.69	**0.001**
**Gender (female)**	− 0.59	− 1.34–0.16	0.12	− 0.71	− 1.40 to − 0.01	**0.046**
**Occupation (doctors)**	− 0.28	− 1.06–0.49	0.46			
**Years of experience (< 4 years)**	1.31	0.61-–1.99	**< 0.001**			
**Primarily work in ED, ICU, or infectious diseases dep. (yes)**	0.81	0.11–1.52	**0.02**	1.05	0.40–1.70	**0.002**
**Experience in working during a previous pandemic (no)**	− 0.45	− 1.33–0.42	0.30			
**Availability of institutional psychological/mental health support (no)**	0.55	− 0.27–1.38	0.189			
**Personal perception of COVID-19 (severe disease)**	0.70	− 0.18–1.58	0.12			
**Worry from contracting COVID-19 at work (severe worry)**	1.66	0.95–2.37	**< 0.001**	0.79	0.11–1.47	**0.024**
**Knowledge score**	− 0.48	− 0.86 to − 0.11	**0.01**	− 0.29	− 0.63–0.06	0.100
**Discrimination intent score**	− 0.05	− 0.13–0.026	0.187			
**Internalized shame scale**	0.78	0.61–0.94	**< 0.001**	0.74	0.57–0.91	**< 0.001**

*ED* emergency department, *ICU* intensive care unit, *COVID-19* coronavirus disease-2019

Bold values: statistically significant at *P* value < 0.05

The adjusted multivariate linear regression model showed that not having a previous experience in working during pandemic (ß = 0.42; *P* = 0.036), high discrimination intention towards COVID-19 patients (as low scores indicate high discrimination intention) (ß = − 0.14; *P* < 0.001) and high perception of stigma resulted from actions of surrounded subjects (ß = 1.08; *P* < 0.001) were significant predictors for perceiving internalized shame among the studied Egyptian HCPs (Table 6).

**Table 6:** Predictors of perceiving internalized shame among the studied health care providers, Egypt (2020)

Variable	Unadjusted			Adjusted (***R***^**2**^ = 0.28)		
	ß	95% CI	***P*** value	ß	95% CI	***P*** value
**Age (< 30 years)**	0.26	− 0.06–0.59	0.11	− 0.21	− 0.51–0.10	0.188
**Gender (female)**	− 0.03	− 0.38–0.32	0.86	− 0.14	− 0.45–0.17	0.376
**Occupation (doctors)**	0.70	0.33–1.06	**< 0.001**	0.33	− 0.02–0.68	0.068
**Experience years (< 4 years)**	0.27	− 0.05–0.60	0.10			
**Primarily work in ED, ICU, or infectious diseases dep. (yes)**	− 0.49	− 0.82 to − 0.16	**0.004**	− 0.15	− 0.48–0.17	0.355
**Experience in working during a previous pandemic (no)**	0.73	0.32-1.14	**< 0.001**	0.42	0.03–0.81	**0.036**
**Availability of institutional psychological/mental health support (no)**	0.23	− 0.15–0.62	0.23			
**Personal expectation about COVID-19 (severe disease)**	0.25	− 0.16–0.66	0.23			
**Worry from contracting COVID-19 at work (severe worry)**	0.97	0.64–1.30	**< 0.001**	0.28	− 0.04–0.61	0.084
**Knowledge score**	− .149	− 0.32–0.02	0.09			
**Discrimination intent score**	− 0.16	− 0.19 to − 0.13	**< 0.001**	− 0.14	− 0.17 to −0.10	**< 0.001**
**Impact stigma score**	0.17	0.13–0.20	**< 0.001**	0.17	0.13–0.201	**< 0.001**

*ED* emergency department, *ICU* intensive care unit, *COVID-19* coronavirus disease-2019

Bold values: statistically significant at *P* value < 0.05

## Discussion

COVID-19 is an emerging highly infectious disease transmitted swiftly from one country to the other. The World Health Organization main concern when declaring the disease as a public emergency was “if the virus spreads to countries with stretched health care system, will they stand it or collapse” [16]. In our study, we present the results of a survey investigating the knowledge, attitude, worry from contracting COVID-19 infection, and its stigma among Egyptian HCPs and analyzing potential risk factors for their perceived worry and stigmatization.

### Knowledge of the disease and attitude towards it

Dealing with a public health emergency as COVID-19 requires that HCPs be aware of the disease, its mode of transmission, diagnosis, prevention, and management protocols. In our study, the average correct answers were very high indicating that Egyptian HCPSs were working with reliable knowledge about the disease in spite of the continuously evolving situation. Our findings suggest that despite false information circulated on social media, Egyptian HCPs were keen to seek the scientific evidence in comparison to other HCPs in the region [17]. Similarly, a systematic review of 20 studies involved 12,072 HCPs assessed the knowledge level about novel Coronavirus-19 during the initial pandemicreported that three fourths of HCPs had good understanding of COVID-19. The study emphasized the role of good knowledge level about COVID-19 disease in improvement of the diagnosis, delaying disease spread, and applying good infection control practices [18].

While nearly three quarters were confident that the pandemic will be eventually controlled globally, less than half were pessimistic that Egypt will have better chances  for  controlling it. This belief stems from the fact that they are aware of the already overburdened health care system in Egypt as a developing country, and to the challenges faced in recruiting, retaining, and protecting sufficient well-trained, supported, and motivated health workers and overcoming any possible shortage of equipment, drugs, and supplies.

### Working conditions and perceptions about the disease

In our study, 40% only of the respondents worked in emergency departments, intensive care units, or infectious diseases departments and the majority had no prior experience with facing pandemics. Nearly one third report that they were exposed to a suspected or confirmed COVID-19 patient. While the majority state that they did not receive specific training on COVID-19 at the time the study was conducted, they still report that they received appropriate guidance on updated procedures for personal safety to be implemented at work. On the contrary, more than half (57%) of Pakistani health care providers reported receiving COVID-19-related training, such as PPE use (donning, doffing), social distancing, isolation, quarantine, and lock-down [19].

Our study highlighted the lack of appropriate institutional mental health support which is a concerning finding in our study. While only one third reported that they were directed to COVID-19-related activities, the majority exhibited changes to their personal protection as routinely wearing face masks, frequent hand hygiene, and practicing social distancing. Another concerning finding our study revealed was that less than one third started wearing scrubs or disposable clothes which was attributed to the shortage in PPEs available. In accordance, the WHO recommends frequent hand hygiene, wearing medical mask, and maintaining social distance as the most effective preventive measures in the community [20]. These positive habit changes were reported in Ethiopia and United Arab Emirates [21, 22].

World Health Organization and Occupational Safety and Health Administration (OSHA) ensured the necessity of provision of personal protective equipment, appropriate training, safe working conditions for the safety of both health worker and patients [23, 24]. However, shortage of personal protective equipment had been experienced by many affected counties regardless of their economic level during the initial coronavirus disease pandemic such as Pakistan, Latin American countries, and the USA [25–27].

Although nearly half exhibited no discrimination intent in treating COVID-19 patients, the majority (80%) expected COVID-19 to be a severe disease with consequent morbidity and mortality; yet, they stated they will continue to work during the pandemic if they are fit and well. The main concern of our study respondents have is contracting COVID-19 infection and transmitting the infection to their loved ones.

At the time of administration of the survey, COVID-19-confirmed patients were referred to assigned isolation hospitals. Establishing social constraints and restricting individuals’ freedoms as working in quarantine or a confinement facility itself can generate fear and incite hysteria consequently and can exacerbate the stigma of the disease [28, 29]. It is logical that these negative emotions and perceived feeling of stigma circulates among HCPS working in these conditions.

### Worry of contracting COVID-19

In pandemics, psychological impacts such as fear and anxiety are natural, especially if the number of infected individuals and death rates is sharply increasing [30]. HCPs not only face overwhelming workload, PPE shortage, paucity of specific drugs, but they are also dealing with an uncertain threatening situation that may cause critical illness or death in addition to moral decisions regarding prioritization of whom to treat within limited resource settings. These circumstances are poised to cause mental discomfort and stir worry among them. In the current study, the majority of the studied HCPs were worried from contracting COVID 19 infections at work (94.7%). In agreement to the current study, predominant proportions of Saudi, Ethiopian and Japanese HCPs felt worry about COVID-19 pandemic [21, 30, 31].

One of the factors that heighten the risk of infection among HCPs and increases their stress is that some patients conceal their contact history with confirmed or suspected COVID-19 patients because of the ongoing stigma related to the disease. The lack of PPEs as scrubs and disposable gowns represents a legitimate driver of worry about contracting the virus. This worry can be lowered if infection control measures are effectively employed**.** Research showed that the psychological effects of quarantine can still be detected months or years later and suggests the need to ensure that effective mitigation measures are put in place as part of the quarantine planning process [32].

Our study demonstrated that expecting COVID-19 to be a severe disease, believing that COVID-19 infection will not be successfully controlled, improbability to continue working during the pandemic even if fit, high discrimination intention towards COVID-19 patients, and perceiving stigma resulted from actions of surrounding subjects were risk factors for increased worry among HCPS to contract the infection while working in these circumstances. Our findings are consistent with recent research demonstrating that COVID-19 pandemic had psychological consequences in the form of negative emotions as fear, anxiety, and depression [33] especially among HCPs [34, 35]. Previous epidemics as severe acute respiratory syndrome (SARS) caused similar significant stress among frontline HCPs [36, 37]. Our findings are consistent with other studies during SARS where emotions experienced by HCPs forced them to resign from their work or perform badly [38].

Our research highlighted that risk factors for severe worry among HCPs were high discrimination intention towards COVID-19 patients and perceiving stigma from actions of surrounding subjects while among risk factors of stigma were high perception of internalized shame from dealing with COVID-19 patients or work in its related field. The findings in our study confirm the mental health burden that Egyptian HCPs experienced at the COVID-19 pandemic. Effective psychological intervention programs for HCPs as assistance hotlines or smart use of social media applications for counselling should be designed [39]**.**

### Stigma perception

Studies from previous epidemics reported that HCPs who were quarantined suffered stigmatization and rejection in their local communities in comparison to those who were not quarantined [32, 38]. A study about the Liberia Ebola epidemic reported that stigma led some quarantined families to be excluded from the community and labelled as dangerous based on being of different ethnicity, tribes, or religion. This behavior led those who fell ill because of any disease to conceal their illness and evade seeking medical help [40]. High stigma perception from work-related COVID-19 exposure was reported among other Egyptian, Indonesian and Nigerian HCPs in the early phase of COVID-19 pandemic [41–43]. In accordance, a study investigated stigmatization against HCPs among non-health care workers. Reported stigmatization responses included the belief in placing severe restrictions on HCWs freedoms and HCPs should be kept in isolation from their communities and their families (more 25% of respondents). Moreover, over a third of American and Canadian respondents avoided HCPs for fear of infection [44].

In the current study, adjusted regression showed that female health care workers were significantly at a higher risk for impact stigma than males. As regards infectious diseases, stigma was reported to be higher in women, and this could be attributed to women are traditionally expected to bear social rejection and negative self-image [45].

Media is a powerful tool in shaping public views and information and has previously contributed to stigmatization in prior outbreaks [32]. Researchers found that social media affected behaviors of risk perception [46]. Since the start of the pandemic, the Egyptian Ministry of Health (MOH) started using sponsored ads on Facebook to educate the public about the disease as a way to use such a powerful tool in its favor [47]. However, with continuous fake news, rumors, and myths concerning COVID-19 easily circulating among the public, Egyptian HCPs became a target for discrimination.

## Study limitations

This study has been conducted during the initial epidemic and quarantine duration in Egypt and before the construction and distribution of COVID-19 vaccines. Moreover, the data was collected using non-probability convenience sample mainly from HCPs in Upper Egypt. It is recommended to conduct national random representative study to evaluate the worry and stigma perception after vaccine availability.

## Conclusions

Considerable levels of worry and stigma perceptions were detected among Egyptian HCPs during the COVID-19 outbreak. The health care system must engage HCPs in effective strategies to improve their mental health and offer psychological support and interventions. Other strategies include designing targeted education campaigns for destigmatizing COVID-19, addressing the public fear, and delivering updated health education initiatives about the disease within the official media outlets.

## Data Availability

The datasets generated and analyzed during the current study are available from the corresponding author on reasonable request.

## Abbreviations

HCPs: Healthcare providers
MOH: Ministry of Health
SARS: Severe acute respiratory syndrome
